# Histone H2A.Z Suppression of Interferon-Stimulated Transcription and Antiviral Immunity Is Modulated by GCN5 and BRD2

**DOI:** 10.1016/j.isci.2018.07.013

**Published:** 2018-07-20

**Authors:** Nancy Au-Yeung, Curt M. Horvath

**Affiliations:** 1 Department of Molecular Biosciences, Northwestern University, Evanston, IL 60208, USA

**Keywords:** Immunology, Immune Response, Transcriptomics

## Abstract

Type I interferon (IFN)-stimulated gene (ISG) expression requires interaction between a transcription factor complex, ISGF3, and target gene promoters to initiate transcription and protection against infection. To uncover chromatin regulatory features of this antiviral immune response, IFN-induced nucleosome and histone dynamics of human ISG loci were examined. ISGF3 recruitment after IFN stimulation was accompanied by nucleosome reorganization at promoters and gene bodies. IFN stimulation induced loss of core histones H2B, H3, and H4, as well as H2A.Z at ISG promoters. A strong correlation was found between H2A.Z occupancy and ISGF3 target sites, and IFN-stimulated H2A.Z removal requires STAT1, STAT2, and IRF9. Neither INO80 nor SWI/SNF participate in IFN-driven H2A.Z eviction, but GCN5 and BRD2 are required. Interference with H2A.Z expression enhanced ISGF3 recruitment to ISG promoters, ISG mRNA expression, and IFN-stimulated antiviral immunity. This indicates that H2A.Z nucleosomes at ISG promoters restrict optimal ISGF3 engagement and modulate the biological response to IFN.

## Highlights

• Type I IFN induces nucleosome reorganization at antiviral target genes
• Histone H2A.Z occupies ISG promoters and is removed following IFN stimulation
• IFN induces H2A.Z remodeling via STAT1, STAT2, IRF9, and cofactors GCN5 and BRD2
• H2A.Z knockdown increases promoter access, ISG transcription, and antiviral response

## Graphical Abstract

**Figure:**
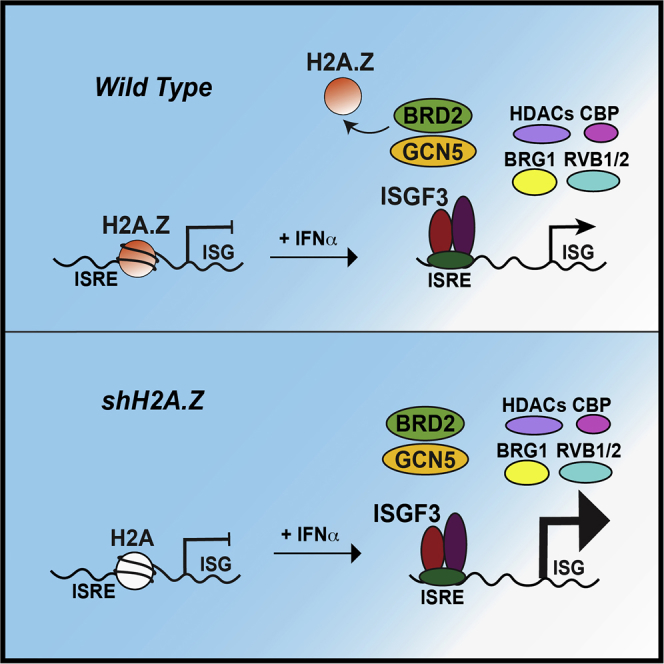

## Introduction

Initially discovered as a soluble antiviral factor secreted from infected cells (Isaacs and Lindenmann, 1957), type I interferon (IFN) is now known to govern a multitude of biological processes related to innate and adaptive immunity, neoplastic transformation (Parker et al., 2016), efficacy of cancer therapies (Zitvogel et al., 2015), and immunomodulatory processes (Gonzalez-Navajas et al., 2012). Most IFN actions are mediated by transcriptional responses that drive the simultaneous expression of hundreds of IFN-stimulated gene (ISG) loci, producing a wide range of products that combine to create a cellular “antiviral state” that prevents virus entry; interferes with cellular and viral RNA transcription, stability, and translation; and thwarts virus replication (Schneider et al., 2014).

IFN actions are largely mediated by a canonical JAK-STAT signaling pathway that induces ISG products (Aaronson and Horvath, 2002, Platanias, 2005). IFN binding to its cell surface receptor engages the tyrosine kinases, JAK1 and TYK2, to phosphorylate latent transcription factors STAT1 and STAT2 on activating tyrosine residues. Phosphorylated STAT1 and STAT2 undergo SH2 domain-mediated dimerization, and associate with an interferon regulatory factor, IRF9, to form the mature ISGF3 transcription factor complex. Nuclear translocation of ISGF3 enables it to bind to IFN-stimulated response element (ISRE) sequences in chromatin, recruit co-activating machinery, and mobilize RNA polymerase II (Pol II) transcription (Au-Yeung et al., 2013, Chang et al., 2004, Stark and Darnell, 2012).

Current evidence indicates that most eukaryotic promoters feature positioned nucleosomes flanking regions that contain regulatory elements for the assembly of transcription regulators, Pol II, and essential or gene-specific co-activators. In addition to nucleosomes composed of an octamer of histones H2A, H2B, H3, and H4 wrapped with ∼147 bp of DNA, less abundant nucleosomes contain histone variants that are associated with specific regulatory phenomena (Buschbeck and Hake, 2017). Histone variants allow for greater control of DNA replication, repair, or transcription and contribute to the efficiency of Pol II elongation, termination, and processivity. Histone H2A.Z is a variant of H2A that is enriched at eukaryotic gene promoters and can also be found at heterochromatin boundaries, at sites of DNA damage repair, and in segregating chromosomes. H2A.Z is frequently associated with active promoters that are marked by H3K4me3 and is thought to function contextually in controlling gene expression through differential co-activator recruitment (Barski et al., 2007, Hu et al., 2013, Ku et al., 2012, Surface et al., 2016). Depending on its post-translational modification, H2A.Z has been implicated as both an activator and inhibitor of transcription regulation and nucleosome stability (Bonisch and Hake, 2012, Ku et al., 2012, Marques et al., 2010, Subramanian et al., 2015, Talbert and Henikoff, 2017). For example, depletion of H2A.Z can result in increased expression of p21 and decreased expression of estrogen receptor target genes in human cells (Gevry et al., 2007, Gevry et al., 2009). Deposition and removal of H2A.Z by chromatin remodeling complexes, SWR1 and INO80, has been inferred from yeast studies, and H2A.Z-specific remodelers continue to be investigated in more complex organisms. H2A.Z is evolutionarily conserved from yeast to human, but its function remains contextual: loss of H2A.Z leads to viable yeast with growth defects, yet it is essential in mouse, *Drosophila*, *Xenopus*, *Tetrahymena*, and *Trypanosoma* (Faast et al., 2001, Zlatanova and Thakar, 2008).

Little is known about how chromatin structure and nucleosome dynamics influence ISGF3 promoter engagement, transcriptional activity, and innate immunity, but several studies have implicated chromatin-remodeling, histone-modifying, and polymerase-activating factors as ISGF3 co-activators (Gnatovskiy et al., 2013, Huang et al., 2002, Kadota and Nagata, 2014, Nusinzon and Horvath, 2003, Patel et al., 2013, Paulson et al., 2002). ISGF3 has been linked to many transcription co-activators that are commonly recruited by the strong STAT2 transcriptional activation domain, often with support from STAT1. Notably, ISGF3 has an absolute requirement for histone deacetylase (HDAC) activity (Nusinzon and Horvath, 2003) for transcriptional stimulation, and STAT2 interacts with HDACs (Chang et al., 2004, Nusinzon and Horvath, 2003, Sakamoto et al., 2004). In addition, ISGF3 engages histone acetyltransferase (HAT) activities from CBP (human CREBBP)/p300 and GCN5 (human KAT2A), and has been linked to SWI/SNF (human BAF) and INO80 chromatin remodeling complexes via specific STAT2 interaction partners including BRG1 (human SMARCA4), RVB1 (human RUVBL1/TIP49), and RVB2 (human RUVBL2/TIP48) (Bhattacharya et al., 1996, Cui et al., 2004, Gnatovskiy et al., 2013, Huang et al., 2002, Liu et al., 2002, Patel et al., 2013, Paulson et al., 2002). HDAC and HAT activity are linked to recruitment of BRD4 to control RNA Pol II elongation through p-TEFb and NELF/DSIF (Patel et al., 2013), and STAT2 association with Mediator subunits and TAF_II_s directly connects ISGF3 to Pol II initiation and elongation machinery (Lau et al., 2003, Paulson et al., 2002). Together the patterns of co-activator and remodeler recruitment are consistent with a highly regulated general and gene-specific ISGF3-mediated transcriptional activation process for ISGs and suggest a role for chromatin structure and composition in antiviral gene regulation.

To investigate ISGF3-mediated interactions with native chromatin, the chromatin architecture and nucleosome organization of ISGs was characterized using chromatin immunoprecipitation sequencing (ChIP-seq) and targeted high-resolution nucleosome position analysis. An IFN-induced nucleosome loss observed at most of the surveyed promoters led to examination of the histone composition at these loci. Evidence is presented here indicating that ISG promoters, like many active eukaryotic promoters, are decorated with the variant histone H2A.Z. This variant nucleosome composition exhibits dynamic behavior in response to IFN stimulation, with loss and recovery coordinated with ISGF3 activity. The loss of H2A.Z requires ISGF3, the HAT, GCN5, and the “bromodomain and extraterminal” (BET) protein BRD2. Interference with H2A.Z enhances ISGF3 recruitment to ISG promoters and hyperactivates ISG expression, leading to greater IFN-induced antiviral protection in cells with H2A.Z depletion. These results reveal dynamic nucleosome remodeling associated with IFN-stimulated transcription and indicate a negative regulatory role for H2A.Z nucleosomes in innate antiviral immune signal transduction.

## Results

### Timing, Occupancy, and Transcriptional Activity of ISGF3 at Target Promoters

**Figure 1:**
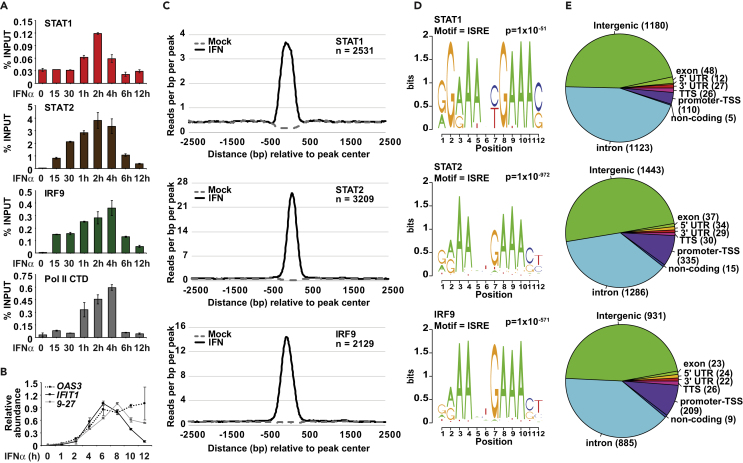
IFN-Stimulated ISGF3 Recruitment and Transcriptional Activity (A) ChIP analysis of IFNα-induced STAT1, STAT2, IRF9, and Pol II C-terminal domain (CTD) recruitment at the *OAS3* promoter locus in HeLa cells after mock treatment (0 min) or IFNα stimulation for 15 min, 30 min, 1 hr, 2 hr, 4 hr, 6 hr, and 12 hr. Error bars denote mean ± SD of three technical replicates. (B) Gene expression analysis of *OAS3*, *IFIT1*/*ISG56,* and *IFITM1/9–27* after mock treatment (0 hr) or 1-, 2-, 4-, 6-, 8-, 10-, or 12-hr IFNα treatment. Relative abundance is normalized to GAPDH. Error bars denote mean ± SD of three technical replicates. (C) Normalized sequencing tag density of mock-treated (dashed) and IFNα-stimulated (solid) STAT1 (top), STAT2 (middle), and IRF9 (bottom) binding at 2,531, 3,209 and 2,129 genomic loci representing sites with a ≥ 2-fold increase in occupancy after IFNα treatment. Tag density is computed 2,500 bp upstream and downstream of the peak center and is grouped into 10 bp bins. (D) DNA sequence logo of the most frequent *de novo* motif identified from 2,531 STAT1 peaks (top), 3,209 STAT2 peaks (middle), and 2,129 IRF9 peaks (bottom) as described in Table S1. For each position, the sequence logo bit height corresponds to its relative frequency within the sequence. The associated motif name and p value are identified above the logo. (E) Distribution of specific annotated DNA (intergenic, intron, promoter-TSS, exon, 5′ UTR, 3′ UTR, non-coding) and the corresponding number of peaks from 2,531 STAT1 peaks (top), 3,209 STAT2 peaks (middle), and 2,129 IRF9 peaks (bottom). See also Table S1.

To establish the foundation for investigating IFN-stimulated ISGF3 occupancy and chromatin dynamics, ChIP time course assays were performed to examine the kinetics of promoter binding by ISGF3 components STAT1, STAT2, and IRF9, as well as Pol II (Figure 1A). Maximal ISGF3 subunits and Pol II recruitment to the ISRE sequence in the *OAS3* promoter was observed between 2 and 4 hr of IFN stimulation and was attenuated after 6 hr (Figure 1A). Parallel samples were analyzed for ISG mRNAs by RT-qPCR, and representative ISGs, *OAS3*, *IFIT1/ISG56,* and *IFITM1/9–27*, achieved a corroborating mRNA increase after 2-hr IFN treatment with peak expression levels between 6 and 8 hr (Figure 1B). These parameters are in agreement with prior studies and serve as a baseline for genome-wide analysis (Hartman et al., 2005, Nusinzon and Horvath, 2003).

To expand these findings, ISGF3 occupancy was examined by ChIP-seq at steady state and after 2-hr IFN stimulation using STAT1, STAT2, and IRF9 antisera. Specific recruitment of ISGF3 was observed throughout the genome following IFN stimulation, with STAT1, STAT2, and IRF9 recruitment to 2,531, 3,209 and 2,129 target loci, respectively (Figure 1C and Table S1). The higher number of STAT2 binding sites is likely due to more efficient precipitation with the STAT2 antibody, but it may also reflect non-canonical IFN-activated factors that include STAT2 (Majoros et al., 2017). Motif analysis revealed the ISRE sequence as the predominant DNA sequence recovered in all datasets, confirming the known affinity of ISGF3 to ISRE elements (Figure 1D). ISGF3 is recruited to diverse intergenic and intronic regions, as well as to well-known ISG promoter-transcription start sites (Figure 1E). Similar to other human transcription factors, IFN-activated STAT1, STAT2, and IRF9 occupy a large number of loci annotated as intergenic and intronic regions (Schmidt et al., 2010). Notably, a greater proportion of STAT2 and IRF9 mapped to TSS loci compared with STAT1 and may reflect the unique and obligatory association of STAT2 and IRF9 in gene regulation (Banninger and Reich, 2004, Lau et al., 2000).

### IFN-Mediated Nucleosome Dynamics at ISGs

**Figure 2:**
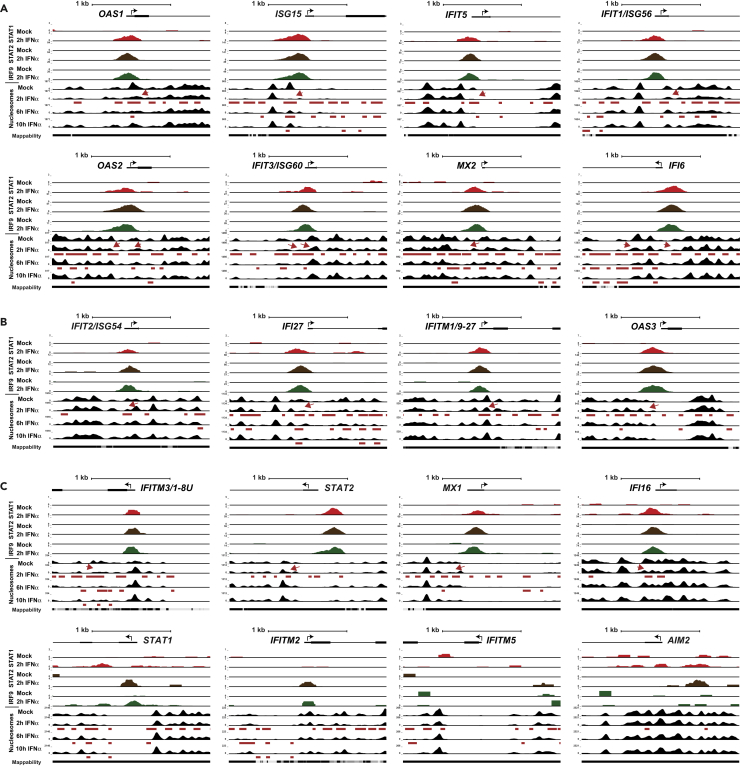
IFN-Stimulated Nucleosome Reorganization Genome browser diagram of IFN-induced ISGF3 recruitment and nucleosome dynamics at select ISGs with (A) high to (B) moderate to (C) low or no nucleosome loss shown within 2000 bp +/− TSS. (A–C) (Top) 5′ End of gene depicted with the black arrow depicting the direction of transcription; the small and large black bars representing untranslated and exonic regions, respectively; and the line representing intronic regions. (Middle) ChIP-seq density of STAT1, STAT2, and IRF9 occupancy after mock or 2-hr IFNα treatment in HeLa cells. (Bottom) Nucleosome occupancy after mock, 2-hr, 6-hr or 10-hr IFNα treatment in HeLa cells. Red arrows highlight nucleosome loss at ISGF3-ISRE proximal regions. Red bars beneath nucleosome maps denote nucleosome loss due to 2, 6, or 10-hr IFNα treatment compared with mock (Poisson p value ≤ 1 × 10^5^). All sequencing reads are normalized to 10 million reads. See also Table S2.

Access to the ISRE requires ISGF3 interaction with the chromatinized ISG promoter. To observe IFN and ISGF3-mediated chromatin dynamics, nucleosome occupancy profiles were determined at select ISGs (Figure 2 and Table S2) throughout a time course of IFN stimulation. A set of 20 ISGs were chosen as representatives of well-documented ISGF3 targets, including members of highly inducible ISG families (e.g., the linked OAS1, 2, and 3 genes [Hovnanian et al., 1998]) and ISGs connected with specific chromatin remodeling machinery such as *IFI27* and *IFITM1/9–27*, genes that exhibit differential dependence on SWI/SNF (BAF) remodeler subunits. Nucleosome occupancy at these loci was surveyed at steady state and after 2, 6, and 10 hr of IFN stimulation (corresponding to times of ISGF3 recruitment, peak transcriptional activity, and attenuation, respectively) using a direct selection micrococcal nuclease method to provide greater sequencing read depth per nucleosome (Freaney et al., 2014, Yigit et al., 2013).

Parallel visualization of the one-dimensional occupancy maps from ChIP-seq and the nucleosome profiles of individual ISGs enables correlations of chromatin changes at sites corresponding to ISGF3 interaction (Figure 2). Comparing mock-treated and IFN-stimulated nucleosome samples over time allows observation of ISGF3 recruitment and corresponding changes to the nucleosome positions. Consistent with contemporary models of nucleosome positioning (Radman-Livaja and Rando, 2010), most of the 20 ISGs were found to have well-positioned nucleosomes in the ISRE region at steady state, with varying degrees of promoter demarcation by nucleosome-depleted regions (NDRs; Figure 2). Gene-specific variations include those with well-positioned +1 and −1 nucleosomes (*IFIT2/ISG54, OAS2*, *IFIT3/ISG60*, *IFI6*, *IFITM3/1-8U*, *MX2*) or +1 and NDR nucleosomes (*OAS1*, *IFIT1*/*ISG56*), those with only +1 nucleosomes (*STAT2*), or those with only −1 nucleosomes (*OAS3*), and genes with no apparent NDR (*IFITM1/9–27*, *IFI16*). In all cases, IFN stimulation resulted in decreased nucleosome positioning strength, with clear disruption over the course of IFN treatment followed by a return to steady state. In most cases, this is evident from a loss of an ISRE-proximal nucleosome (arrows in Figure 2). To quantify the nucleosome loss, the Dynamic Analysis of Nucleosome Positioning and Occupancy Software (DANPOS; [Chen et al., 2013]) was used to indicate changes to nucleosome positions with a p value ≤ 1 × 10^−5^ following IFN stimulation. The normalized tag counts between mock-treated and IFN-treated samples confirmed statistically significant nucleosome loss at ISG promoters that propagated over time throughout the gene bodies (red bars in Figure 2). These findings are not dissimilar to ATAC-seq data indicating that IFN stimulation of B cells increased chromatin accessibility at the TSS of ISGs (Mostafavi et al., 2016). Within the 20 ISGs examined here, the most prominent chromatin alterations coincided with strong ISGF3 peaks and well-positioned nucleosomes (i.e., OAS1), but not strictly at the TSS (i.e., ISG15).

### IFN-Induced Histone Dynamics at ISG Promoters

**Figure 3:**
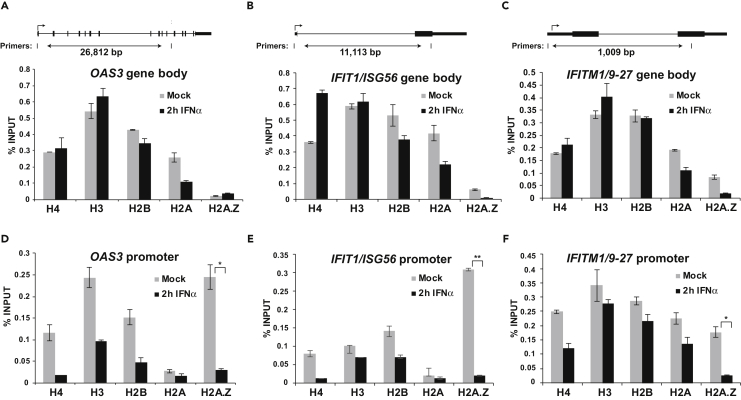
IFN-Stimulated Loss of Histones H2A.Z, H2B, H3, and H4 at ISG Promoters (A–F) ChIP analysis of histones H4, H3, H2B, H2A, and H2A.Z occupancy at the gene body (A–C) or gene promoter (D–F) of *OAS3*, *IFIT1/ISG56*, *IFITM1/9–27* during steady state and after 2-hr IFNα stimulation. The position of the gene body and promoter-specific primers and their relative distance are indicated in the upper panel of A–C. Error bars denote mean ± SD of three technical replicates from one representative experiment. Statistical analysis was computed using the Student's t test with n ≥ 2 (*p < 0.05, **p < 0.005).

To investigate the potential mechanisms underlying nucleosome dynamics at ISGs, the presence of core histones was examined using ChIP-qPCR and primers specific to either the ISRE region of the promoter or distal regions of the gene bodies of *OAS3*, *IFIT1/ISG56*, and *IFITM1/9–27* (Figure 3 and Table S3). In the gene bodies, all four core histones (H2A, H2B, H3, and H4) were present at steady state and remained relatively constant following a 2-hr IFN treatment (Figures 3A–3C). In contrast, the core histones H2B, H3, and H4 were readily detected at ISG promoters, whereas H2A was notably underrepresented. Instead, the histone variant H2A.Z was detected at the ISG promoters (Figures 3D–3F). Stimulation with IFN decreased promoter-associated histones, observed most dramatically for H2A.Z, consistent with the observed nucleosome reorganization identified at ISG promoters (Figure 2). H2A.Z is well known for its association with the promoter TSS region and colocalization with specific histone modification marks, including H3K4me3, in a variety of biological systems, including genes that respond to environmental stimuli (Barski et al., 2007, Hu et al., 2013, Ku et al., 2012). As such, the presence of H2A.Z at ISG promoters provides a tractable system for investigating H2A.Z dynamics and biological impact in mammalian cells.

### H2A.Z Is Inversely Correlated with ISGF3 Recruitment

**Figure 4:**
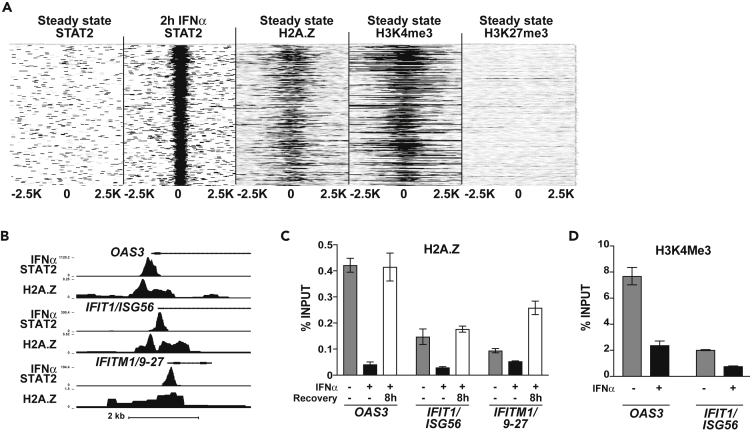
Histone Variant, H2A.Z, Is a Dynamic Component of ISG Promoters (A) Heatmap depicting steady-state and IFNα-recruited STAT2 occupancy (in-house ChIP-seq) compared with steady-state H2A.Z, H3K4me3, and H3K27me3 occupancy (ENCODE ChIP-seq) at the top 250 enriched STAT2 target loci spanning ±2,500 bp from the STAT2 peak center. H2A.Z and STAT2 occupancy at these loci had a Pearson correlation coefficient of R^2^ = 0.83. (B) Genome browser view of H2A.Z occupancy at steady state and STAT2 occupancy after 2-hr IFNα stimulation at three ISGs, *OAS3*, *IFIT1/ISG56,* and *IFITM1/9–27*. (C) ChIP analysis of H2A.Z removal and recovery after 3-hr IFNα treatment followed by recovery without IFNα for 0 hr or 8 hr at the *OAS3*, *IFIT1/ISG56*, and *IFITM1/9–27* promoters. Error bars denote mean ± SD of a representative experiment with three technical replicates. (D) ChIP analysis of steady-state and IFN-stimulated H3K4me3 at *OAS3* and *IFIT1/ISG56* promoters. Error bars denote mean ± SD of a representative experiment with three technical replicates. See also Table S1.

The previously unrecognized association of H2A.Z with ISG promoters suggested that this histone variant might be a more general feature of ISGF3 target genes. To test this idea, a HeLa cell H2A.Z ChIP-seq dataset (ENCODE Project Consortium, 2012; Rosenbloom et al., 2013) was compared to the top 250 IFN-induced STAT2 targets (Figure 4A). A clear correlation was found between the top IFN-activated target genes and H2A.Z occupancy at steady state (R^2^ = 0.83), and examination of target promoter regions indicates that peaks of H2A.Z deposition closely overlap with STAT2-binding ISRE sites (Figure 4B). The presence of H2A.Z inversely correlates with IFN stimulation, and this relationship is further verified by IFN stimulation and recovery experiments. H2A.Z is lost from ISG promoters, whereas ISGF3 is active, but recovers by 8 hr post-stimulation (Figure 4C). H2A.Z has been shown to colocalize with H3K4me3 and at bivalent promoters containing both H3K4me3 and H3K27me3 marks (Ku et al., 2012). Consistent with this observation, the top STAT2 targets also bear the active mark H3K4me3 (Figure 4A), which is coordinately lost from ISGs, *OAS3* and *IFIT1/ISG56,* following IFN stimulation (Figure 4D).

**Figure 5:**
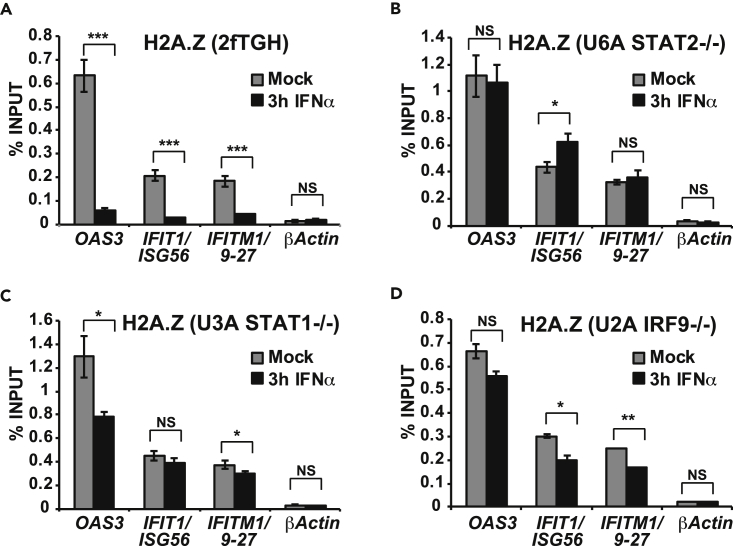
H2A.Z Removal Requires ISGF3 (A) ChIP analysis of H2A.Z in 2fTGH cells with intact ISGF3 at the promoter region of *OAS3*, *IFIT1/ISG56,* and *IFITM1/9–27* with mock or 3-hr IFNα treatment. Error bars denote mean ± SD of one representative experiment with three technical replicates. Statistical analysis was computed using the Student's t test with n ≥ 2 (*p < 0.05, **p < 0.005, ***p < 0.0005, NS, not significant). (B) Same as A but with STAT2-deficient U6A cells. (C) Same as A but with STAT1-deficient U3A cells. (D) Same as A but with IRF9-deficient U2A cells.

To determine whether IFN-stimulated H2A.Z dynamics require ISGF3 activity, H2A.Z loss was examined in a series of cell lines with single gene defects in ISGF3 components STAT1, STAT2, or IRF9 (John et al., 1991, Leung et al., 1995, McKendry et al., 1991). In the IFN-responsive parent 2fTGH cells, H2A.Z localized at ISG promoters and was lost following IFN stimulation (Figure 5A). In contrast, in the daughter cell lines U3A, U6A, and U2A, with defects in either STAT1, STAT2, or IRF9, H2A.Z remained at ISG promoters following stimulation, indicating that ISGF3 is required for efficient H2A.Z removal (Figures 5B–5D).

### INO80 and SWI/SNF Do Not Alter IFN-Induced H2A.Z Removal

Several histone and chromatin-modifying activities have been linked to transcriptional activation by IFN signaling and ISGF3, including HATs, HDACs, and the remodeling machines related to SWI/SNF (BAF) and INO80 (Bhattacharya et al., 1996, Chang et al., 2004, Cui et al., 2004, Gnatovskiy et al., 2013, Huang et al., 2002, Liu et al., 2002, Nusinzon and Horvath, 2003, Patel et al., 2013, Paulson et al., 2002, Sakamoto et al., 2004). In lower eukaryotes, homologous machinery has been implicated in H2A.Z deposition and removal; the yeast chromatin remodeling complexes SWR1 and INO80 have been implicated in H2A.Z deposition and removal (Mizuguchi et al., 2004, Yen et al., 2013), and H2A.Z and SWI/SNF are thought to be partially redundant in yeast, where deletion of H2A.Z increases the need for SWI/SNF (Santisteban et al., 2000). In mammals, the RVB1 (RUVBL1) and RVB2 (RUVBL2) proteins that are subunits of BAF, INO80, SWR1 (SRCAP), and TIP60 complexes (Huen et al., 2010) were found to cooperate with STAT2 and regulate ISGF3 transcriptional activity, but through an unknown mechanism (Gnatovskiy et al., 2013). SWI/SNF (BAF) can remodel the chromatin structure of the ISGs, *IFITM1/9–27* and *IFITM3/1-8U* (Cui et al., 2004, Liu et al., 2002), and the ATPase subunit, BRG1 (SMARCA4), is required for a subset of ISG transcription (Huang et al., 2002).

**Figure 6:**
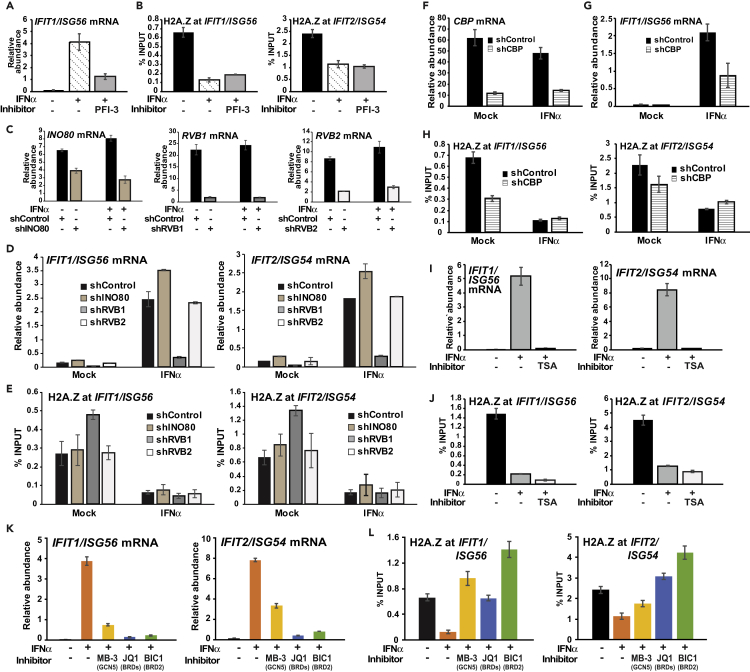
GCN5 and BRD2 Are Essential to IFN-Induced H2A.Z Loss (A and B) HeLa cells were mock-treated or IFNα-treated with or without PFI-3 for 3 hr, and then analyzed for (A) *IFIT1/ISG56* mRNA expression by RT-qPCR and (B) ChIP assays of H2A.Z occupancy at *IFIT1/ISG56* and *IFIT2/ISG54* promoters. Error bars denote mean ± SD of one representative experiment with technical triplicates. (C–E) HeLa cells were transduced with shRNA vectors targeting *INO80*, *RVB1*, *RVB2,* or control. (C) Expression of shRNA targets in mock and 3-hr IFNα-treated cells was measured by RT-qPCR. (D) *IFIT1/ISG54* and *IFIT2/ISG56* mRNA expression in mock and 3-hr IFNα-treated cells harboring the indicated shRNA was measured by RT-qPCR. (E) ChIP assay of H2A.Z occupancy at *IFIT1/ISG56* and *IFIT2/ISG54* promoters in mock and 2-hr IFNα-treated cells containing the indicated shRNA target. Error bars denote mean ± SD of three biological replicates and three technical replicates. For siRNA knockdown of *SRCAP*, see Figure S1. (F–H) HeLa cells were transduced with *CBP* shRNA or control shRNA. (F) Expression of shRNA target in mock and 3-hr IFNα-treated cells was measured by RT-qPCR. (G) *IFIT1/ISG56* mRNA expression in mock and 3-hr IFNα-treated cells harboring the indicated shRNA was measured by RT-qPCR. (H) ChIP assay of H2A.Z occupancy at *IFIT1/ISG56* and *IFIT2/ISG54* promoters in mock and 2-hr IFNα-treated cells containing the indicated shRNA target. Error bars denote mean ± SD of a representative experiment with technical triplicates. (I and J) HeLa cells were mock-treated or IFNα-treated with or without trichostatin A (TSA) for 3 hr and then analyzed for (I) *IFIT1/ISG56* and *IFIT2/ISG54* mRNA expression and (J) H2A.Z occupancy at *IFIT1/ISG56* and *IFIT2/ISG54* promoters. Error bars denote mean ± SD of one representative experiment with technical triplicates. (K and L) HeLa cells were pretreated with MB-3 or BET inhibitors JQ1 and BIC1 for 1 hr, mock-treated or stimulated with IFNα for 3 hr (+/− inhibitor), then analyzed for (K) *IFIT1/ISG56* and *IFIT2/ISG54* mRNA expression and (L) H2A.Z occupancy at *IFIT1/ISG56* and *IFIT2/ISG54*. Error bars denote mean ± SD of one representative experiment with technical triplicates. See also Figures S1–S3.

To determine the machinery involved in IFN-induced, ISGF3-dependent H2A.Z loss, these transcriptional cofactors were examined using chemical inhibitor-, small interfering RNA (siRNA)-, or short hairpin RNA (shRNA)-based approaches (Figures 6 and S1). Interference with SWI/SNF complex with the BRG1/BRM inhibitor, PFI-3, potently inhibited ISG activation (Figure 6A). However, this treatment did not alter IFN-dependent H2A.Z removal (Figure 6B). To target the INO80 complex, shRNA was used to knockdown the INO80 subunit and its associates, RVB1 and RVB2 (Figure 6C). INO80 and RVB2 interference had little effect on IFN-induced mRNA expression, whereas RVB1 interference effectively prevented ISG transcription (Figure 6D). Examination of H2A.Z occupancy revealed that none of these INO80 complex proteins were required for H2A.Z removal following IFN stimulation, although it is interesting to note that RVB1 shRNA led to greater H2A.Z ChIP signals at steady state (Figure 6E). Analysis of the SWR1 (SRCAP) complex, which deposits H2A.Z (Mizuguchi et al., 2004), demonstrated that knocking down the expression of the ATPase subunit, SRCAP, led to an unexpected increase in steady-state H2A.Z occupancy, but was insufficient to inhibit IFN-induced H2A.Z removal (Figure S1). These experiments rule out the SWI/SNF (BAF), SWR1 (SRCAP), and INO80 complexes in the process of IFN-mediated H2A.Z removal.

### GCN5 and BRD2 Are Essential to IFN-Induced H2A.Z Loss at ISGs

Both HAT and HDAC activities are required for positive regulation of ISG transcription. Two HATs, GCN5 (KAT2A) and CBP (CREBBP), interact with ISGF3 and participate in ISG transcription (Bhattacharya et al., 1996, Paulson et al., 2002). HDACs are also essential for the regulation of ISG transcription (Chang et al., 2004, Nusinzon and Horvath, 2003, Sakamoto et al., 2004). Targeting CBP by lentiviral shRNA (Figure 6F) interfered with ISG transcription (Figure 6G) and reduced steady-state H2A.Z levels at ISG promoters, but H2A.Z was still removed after IFN stimulation (Figure 6H). Likewise, treatment with the HDAC inhibitor trichostatin A potently inhibited ISGF3 transcriptional activity (Figure 6I), but no effect was observed for H2A.Z in ChIP assay (Figure 6J), ruling out class I and II HDACs in this process. In contrast, inhibition of GCN5 with the compound MB-3 not only downregulated ISG transcription (Figure 6K) but also abrogated IFN-induced H2A.Z loss (Figures 6L and S2A). Similar inhibition of IFN-induced H2A.Z loss by inhibiting GCN5 activity is observed in 2fTGH cells (Figures S2B and S2C).

Histone H3 and H4 acetylation levels have been shown to be altered in response to IFN stimulation (Nusinzon and Horvath, 2003, Paulson et al., 2002), and H2A.Z dynamic regulation is also under acetylation control (Sevilla and Binda, 2014; see Figure S3). Bromodomains in the BET family proteins, BRD2, BRD3, BRD4, and BRDT, recognize and bind to acetylated lysine residues on histones to execute histone chaperone activities and recruit transcriptional machinery (Taniguchi, 2016). In the IFN response, BRD4 is an adaptor used for the recruitment of pTEFb and NELF/DSIF for ISG transcription elongation (Patel et al., 2013), and although BRD2 has not previously been examined for a role in the IFN system, it has been shown to preferentially associate with H2A.Z-containing nucleosomes rather than H2A and its recruitment has been linked to H2A.Z and acetylated H4K12 (Draker et al., 2012). Both the BET inhibitor, JQ1 (targeting BRD4 and BRD2) and BIC1 (a more selective BRD2 inhibitor), were able to interfere with ISG transcription (Figure 6K) and prevent IFN-stimulated H2A.Z removal (Figures 6L and S2) including acetylated H2A.Z, which is known to be enriched at active genes (Figure S3) (Ku et al., 2012). The inhibitory effects of BET inhibitors on IFN-induced H2A.Z removal were also observed in 2fTGH cells, indicating it is a general feature of ISG regulation (Figures S2B and S2C). Together, these results demonstrate that GCN5 HAT and BRD2 bromodomain-binding activity are required to regulate IFN-induced H2A.Z removal.

### Loss of H2A.Z Enables Greater ISGF3 Recruitment

**Figure 7:**
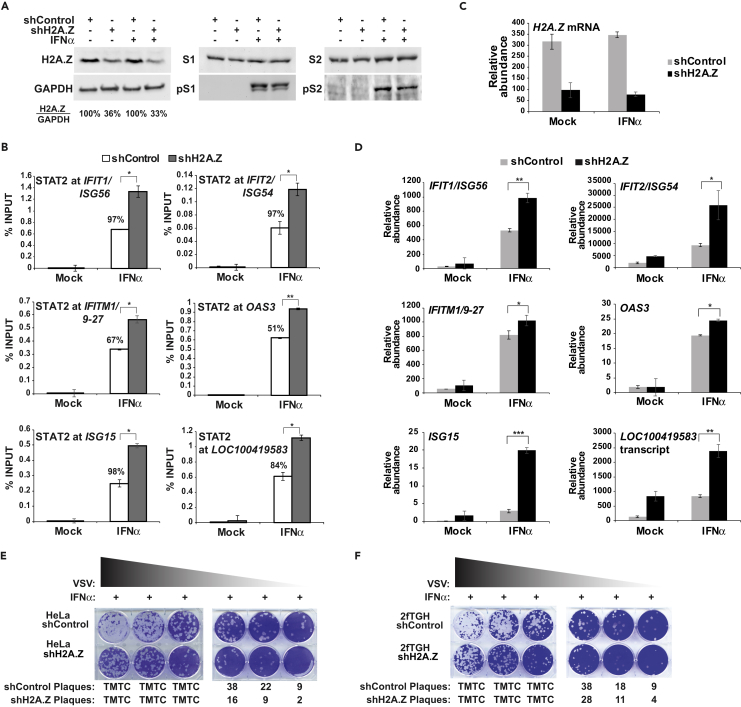
H2A.Z Suppresses ISGF3 Occupancy, ISG Expression, and IFN-Mediated Antiviral Protection HeLa cells were transduced with an shRNA vector targeting H2A.Z or a non-targeting control. (A) Immunoblot of H2A.Z, STAT1, phosphotyrosine 701 STAT1, STAT2, phosphotyrosine 690 STAT2, and GAPDH protein expression in control or H2A.Z knockdown HeLa cells with or without 1-hr IFNα treatment. H2A.Z expression level normalized to GAPDH indicated as % of control. (B) ChIP analysis of STAT2 occupancy in H2A.Z knockdown or control HeLa cells with or without 1-hr IFNα stimulation at promoters of *IFIT1/ISG56*, *IFIT2/ISG54*, *IFITM1/9–27*, *OAS3*, *ISG15*, and *LOC100419583*. % Indicates the increased percentage of STAT2 occupation in shH2A.Z cells compared with non-targeting control cells. Error bars denote mean ± SD of a representative experiment with technical triplicates. Statistical analysis was computed using Student's t test with n ≥ 2 (*p < 0.05, **p < 0.005.). (C) H2A.Z mRNA levels were quantified by RT-qPCR in unstimulated and 10-hr IFNα-stimulated H2A.Z knockdown or control cells. Error bars denote mean ± SD of a representative experiment with technical triplicates. (D) Levels of ISG mRNAs, *IFIT1/ISG56*, *IFIT2/ISG54*, *IFITM1/9–27*, *OAS3*, *ISG15*, and *LOC100419583* were measured as in (C). Statistical analysis was computed using Student's t test with n ≥ 3 (*p < 0.05, **p < 0.005, ***p< 0.0005). (E) Plaque assay in HeLa cells harboring control shRNA or H2A.Z shRNA. Cells were treated for 9 hr with IFNα, followed by 1.5 hr inoculation with a titration of vesicular stomatitis virus (VSV), and then overlaid with DMEM-agar at 37°C for 72 hr before staining with crystal violet. TMTC, too many to count. (F) Same as E, but in control or H2A.Z-deficient 2fTGH cells. See also Figure S4.

The uniform decoration of ISG promoters with H2A.Z, and its IFN-induced loss and recovery, suggested a potential role for H2A.Z in ISG regulation and biological activity. To investigate the impact of H2A.Z in IFN responses and ISG transcription, knockdown experiments were conducted in cells harboring shRNA against H2A.Z or a non-silencing control sequence. H2A.Z-shRNA reduced H2A.Z protein levels by 64%–67% (e.g., Figure 7A). ChIP assays determined that depletion of H2A.Z resulted in increased levels of STAT2 occupancy at the ISG promoters after IFN stimulation compared with the control cells (Figure 7B), resulting in a 51%–98% increase in ChIP signals at individual ISG loci. Similar levels of total and tyrosine-phosphorylated STAT1 and STAT2 were found in control and knockdown cells, confirming that H2A.Z knockdown did not alter IFN-JAK-STAT signaling (Figure 7A). These results indicate H2A.Z-containing nucleosomes restrict maximal IFN-induced ISGF3 occupancy at ISG promoters.

### Loss of H2A.Z Increases ISG Expression

The increased ISGF3 occupancy observed in H2A.Z-shRNA cells suggested the possibility of altered ISG transcription. *H2A.Z* mRNA levels did not change due to IFN stimulation, indicating it is not itself an ISG, and shRNA expression resulted in a significant decrease in *H2A.Z* mRNA (70%–78%) (Figure 7C). ISG mRNA levels were measured by RT-qPCR in H2A.Z-shRNA and control cells (Figure 7D). For all loci tested, increased ISG mRNA levels (2- to 6-fold) were observed in H2A.Z-shRNA cells compared with control cells. The increase in ISGF3 occupancy due to H2A.Z deficiency results in increased ISG mRNA expression.

### Loss of H2A.Z Enhances the IFN-Stimulated Antiviral Response

The IFN-stimulated transcriptional response is the primary cell-autonomous innate antiviral response that inhibits virus replication. To test the overall phenotypic impact of H2A.Z deficiency in the IFN response, a biological response assay was used to assess a role for H2A.Z in IFN-induced antiviral protection. H2A.Z-shRNA and control shRNA cells were stimulated with IFN for 9 hr to establish an antiviral state, then challenged with vesicular stomatitis virus (VSV) infection, overlayed with agarose, and viral plaques quantified. Virus replication was virtually identical in H2A.Z-shRNA or control shRNA cells in the absence of IFN stimulation, irrespective of H2A.Z depletion (Figure S4). In contrast, IFN-mediated virus interference was clearly increased in the H2A.Z-shRNA cells compared with control cells, resulting in 2×–5× fewer plaques in the absence of H2A.Z in HeLa (Figure 7E) and 2fTGH cells (Figure 7F). This increased antiviral protection observed under reduced H2A.Z conditions is consistent with increased ISGF3 recruitment and ISG transcription and supports the conclusion that H2A.Z acts as a negative regulator of antiviral responses in human cells.

## Discussion

To better understand the contribution of chromatin and nucleosome dynamics to mammalian antiviral transcriptional regulation, the chromatin architecture and nucleosome organization of ISG loci were characterized to correlate IFN-mediated changes with the activation of ISGF3 components, STAT1, STAT2, and IRF9. Overall, stable steady-state nucleosome positions at ISG loci were rearranged by IFN stimulation, giving rise to a transient alteration in chromatin structure during the response. These alterations were particularly evident at ISGF3-ISRE promoter regions, supporting the notion that ISGF3-mediated recruitment of chromatin-modifying enzymes serves to remodel chromatin.

Direct examination of the histone composition of ISG promoters not only confirmed the IFN-induced nucleosome loss but also identified an absence of histone H2A. In its place, the H2A variant H2A.Z was found to be enriched at or near the ISRE regions of most highly responsive ISGs before IFN stimulation. H2A.Z presence at ISG promoters was found to be tightly but inversely correlated with IFN-stimulated STAT2 occupancy, and IFN stimulation induced acute and transient loss of H2A.Z at ISG promoters coinciding with the cycle of ISGF3 activation, inactivation, and transcription attenuation. H2A.Z removal requires ISGF3 components STAT1, STAT2, or IRF9, indicating a role for ISGF3 in recruiting and coordinating machinery for H2A.Z nucleosome eviction. Identification of H2A.Z at ISG promoters is in agreement with the general paradigm of H2A.Z nucleosome association with active or inducible gene promoters (Barski et al., 2007, Hu et al., 2013, Raisner et al., 2005). Here, H2A.Z colocalizes at steady state with the active histone modification mark, H3K4me3, but not the repressive H3K27me3 mark, and H3K4me3 is reduced after IFN. Additional studies will be required to examine histone modifications that are present before or following IFN stimulation and their correlation with H2A.Z chromatin dynamics at ISG promoters.

To determine the factor(s) regulating H2A.Z loss, we examined a broad array of co-activators that were known to be associated with ISGF3 and ISG transcription, as well as those implicated previously in H2A.Z deposition or removal from other systems. In lower eukaryotes, SWR1 (SRCAP), was demonstrated to deposit H2A.Z, and knockdown of mammalian SRCAP did not alter IFN-induced H2A.Z removal. Although the INO80 remodeling complex is purported to be responsible for removing H2A.Z nucleosomes in lower eukaryotes (Lai and Pugh, 2017), results indicate that neither RVB nor the INO80 components are necessary for IFN-induced H2A.Z removal in mammalian cells. Unexpectedly, interference with INO80 or RVB2 had no discernable effect on ISG transcription, although it is possible that RNAi was insufficient to deplete stable protein activity. In contrast, RVB1, as well as CBP, BRG1/BRM, and HDACs were all found to be essential for ISG mRNA transcription, but their inhibition had no effect on IFN-stimulated H2A.Z removal. These proteins are otherwise required for ISG transcription, acting either through another remodeler such as SWI/SNF (BAF) or through distinct mechanisms. The increase of steady-state H2A.Z in SRCAP and RVB1 knockdown cells suggests that the human SRCAP subunit/complex differs from its yeast homolog, which may not be surprising given the fact that the interferon/JAK-STAT system does not exist in yeast.

Instead, the HAT GCN5 was identified as being required for ISG transcription and as an essential component of H2A.Z eviction. Inhibition of GCN5 using MB-3 generally inhibited the IFN-induced H2A.Z removal at the ISG promoters examined in both HeLa and 2fTGH cell lines, but a smaller effect of GCN5 inhibition was observed at the *IFIT2/ISG54* promoter. We postulate that this might indicate redundancy in HAT activities or reflect heterogeneity at individual ISG loci. GCN5 or GCN5-containing HAT complexes have been shown to acetylate histones H3, H4, and H2A.Z, and these acetylated lysines are in turn recognized by BET family protein bromodomains (Anamika et al., 2010, Millar et al., 2006). The BET family protein BRD4 is a mediator of ISG transcriptional elongation by recruiting pTEFb and NELF/DSIF to paused polymerases (Patel et al., 2013), and we find that BRD2 inhibition prevents both ISG transcription and H2A.Z removal. This finding is consistent with BRD2's preferential association with H2A.Z-containing nucleosomes (Draker et al., 2012, Punzeler et al., 2017, Surface et al., 2016).

H2A.Z is thought to influence nucleosome stability and positioning, and consequently alter the ability of activating or repressing factors to make stable or transient contact with DNA. This general property of H2A.Z nucleosomes can result in both positive and negative regulation, depending on gene-specific, tissue-specific, and/or context-specific transcriptional responses (Marques et al., 2010, Subramanian et al., 2015). The physical and regulatory properties of H2A.Z-containing nucleosomes have been widely studied, but the literature reflects a variety of roles. H2A.Z has been associated with both transcription activation and transcription inhibition, and has been described as both an activator and repressor of gene expression (Hu et al., 2013, Ku et al., 2012, Schones et al., 2008, Surface et al., 2016, Zlatanova and Thakar, 2008). For example, in embryonic stem cell differentiation H2A.Z is important for facilitating the recruitment of chromatin activators and repressors (Hu et al., 2013, Surface et al., 2016). Although knocking down H2A.Z does not alter steady-state ISG expression, loss of H2A.Z nucleosomes allows ISGF3 greater access to DNA, increases ISG expression, and produces a more effective innate antiviral response. Altogether, these results suggest a model wherein ISGF3 recruits GCN5 to acetylate histones, leading to BRD2 engagement, and to mediate remodeling/eviction of H2A.Z nucleosomes. Reduced H2A.Z relieves the need to remodel the nucleosomes at ISG promoters, enabling ISGF3 to bind and activate ISG expression more easily. This greater access translates into more potent antiviral activity.

Regulating ISG transcription is critical for cellular antiviral responses and for subsequent immune responses, and chronic IFN signaling can lead to inflammatory and autoimmune diseases (Rodero and Crow, 2016). Dysregulation of ISG transcription is also observed in tumors and contributes to immunotherapy resistance (Benci et al., 2016). The combinatorial use of BET inhibitors or other epigenetic drugs with immunotherapy is a current strategy to improve treatment outcomes (Marazzi et al., 2018), and H2A.Z expression is also associated with malignancies (Monteiro et al., 2014), suggesting an interrelated regulatory network that includes cytokine-activated transcription, nucleosome dynamics, and chromatin remodeling activities that can be exploited for augmenting therapeutic strategies.

## Methods

All methods can be found in the accompanying Transparent Methods supplemental file.

## Data and Software Availability

All deep sequencing data have been deposited in GEO under accession number GSE110067.

## Supplemental Information

Document S1. Transparent Methods, Figures S1–S4, and Tables S2 and S3

Table S1. ChIP-Seq Results of STAT1, STAT2, and IRF9 Reads, Related to Figure 1 Sequence reads of STAT1, STAT2, and IRF9 ChIP-seq from HeLa cells either mock-treated or IFNα-treated for 2 hr. Peaks were generated from MACS analysis, and differential ChIP-seq peaks from IFNα-treated STAT1, STAT2, and IRF9 are annotated.
