# Supplementary material for: Histone H2A.Z Suppression of Interferon-Stimulated Transcription and Antiviral Immunity Is Modulated by GCN5 and BRD2
Source: iScience. 2018 Jul 20;6:68–82. doi: 10.1016/j.isci.2018.07.013 (PMC6137307; doi:10.1016/j.isci.2018.07.013)
Supplement: Document S1. Transparent Methods, Figures S1–S4, and Tables S2 and S3 [file mmc1.pdf]

ISCI, Volume *6*

Supplemental Information

Histone H2A.Z Suppression of Interferon-Stimulated Transcription and Antiviral Immunity Is Modulated by GCN5 and BRD2

Nancy Au-Yeung and Curt M. Horvath

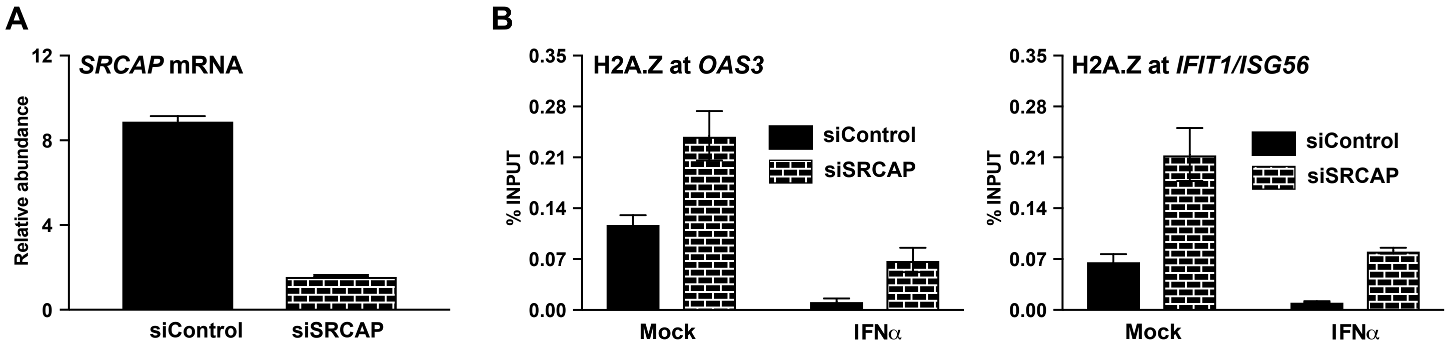

Figure S1. SRCAP is not required for IFN-induced H2A.Z loss, Related to Figure 6
(A-B) HeLa cells were transfected with siRNA against control (non-targeting sequence) or SRCAP. (A) *SRCAP* mRNA expression in control or SRCAP knockdown cells and normalized to GAPDH. (B) ChIP assay of H2A.Z occupancy at *OAS3 and IFIT1/ISG56* promoters in control or *SRCAP* knockdown cells after mock or 3h IFNα-treated cells. Error bars denote mean ± SD of a representative experiment with technical triplicates.

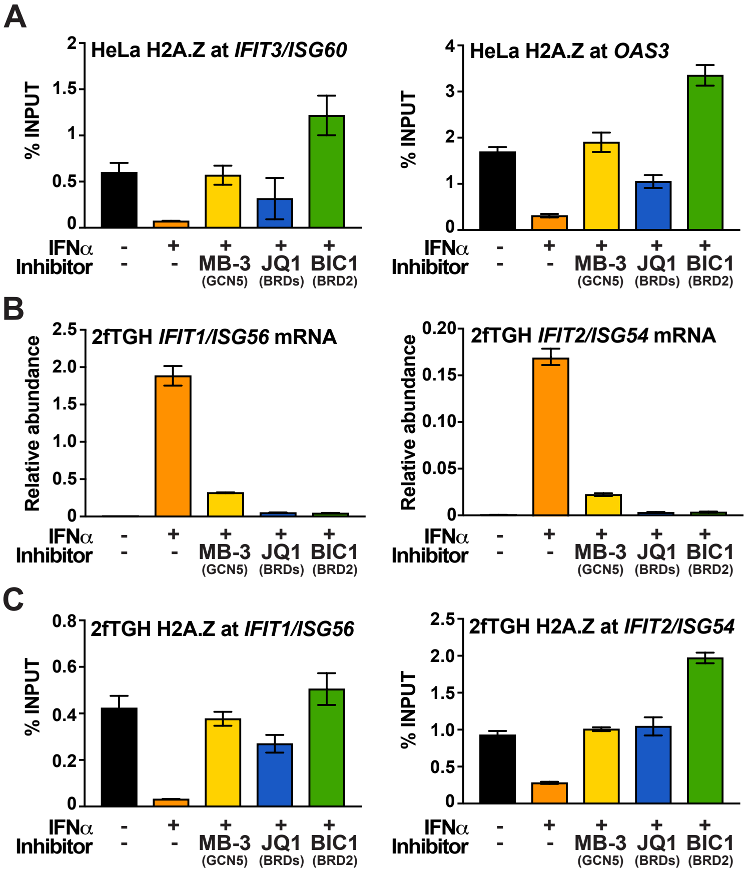

Figure S2. GCN5 and BRD2 are essential to IFN-induced H2A.Z loss, Related to Figure 6
(A) HeLa cells were pretreated with MB-3 or BET inhibitors JQ1 and BIC1 for 1h, mock-treated or stimulated with IFNα for 3h (+/- inhibitor), then analyzed for H2A.Z occupancy at *IFIT3/ISG60* and *OAS3*. Error bars denote mean ± SD of a representative experiment with technical triplicates. (B-C) 2fTGH cells were pretreated with MB-3 or BET inhibitors JQ1 and BIC1 for 1h, mock-treated or stimulated with IFNα for 3h (+/- inhibitor), then analyzed for (B) *IFIT1/ISG56* and *IFIT2/ISG54* mRNA expression and (C) H2A.Z occupancy at *IFIT1/ISG56* and *IFIT2/ISG54*. Error bars denote mean ± SD of a representative experiment with technical triplicates.

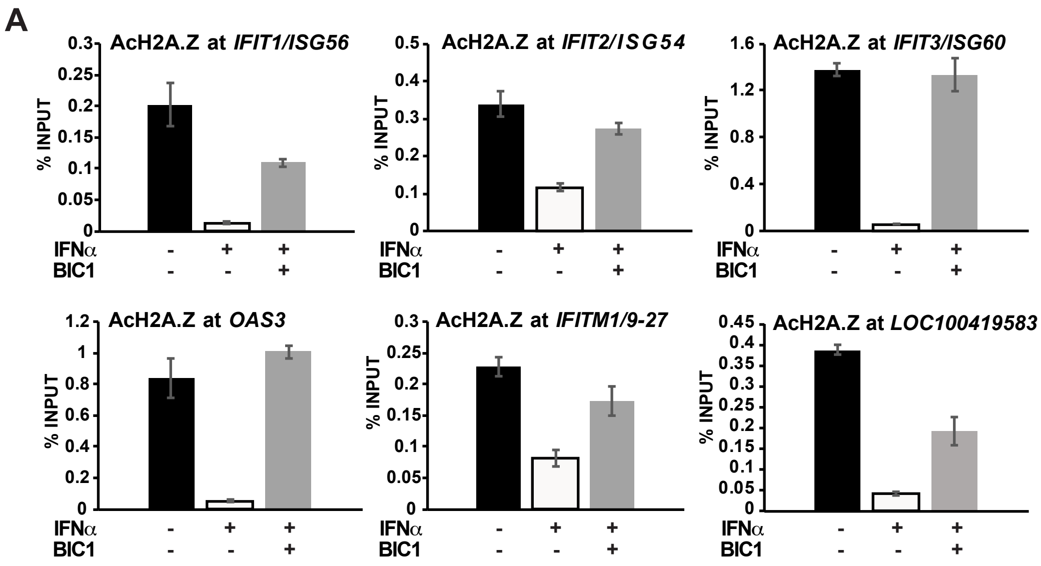

Figure S3. IFN-stimulated acetylated H2A.Z loss requires BRD2, Related to Figure 6

(A) ChIP analysis of acetylated H2A.Z (K4, K7, K11) after mock, 3h IFNα or 1h pre-incubation with BIC1 followed by 3h IFNα/BIC1 treatment at the gene promoters of *IFIT1/ISG56*, *IFIT2/ISG54*, *IFIT3/ISG60*, *OAS3*, *IFITM1/9-27* and *LOC100419583*. Error bars denote mean ± SD of a representative experiment with three technical replicates.

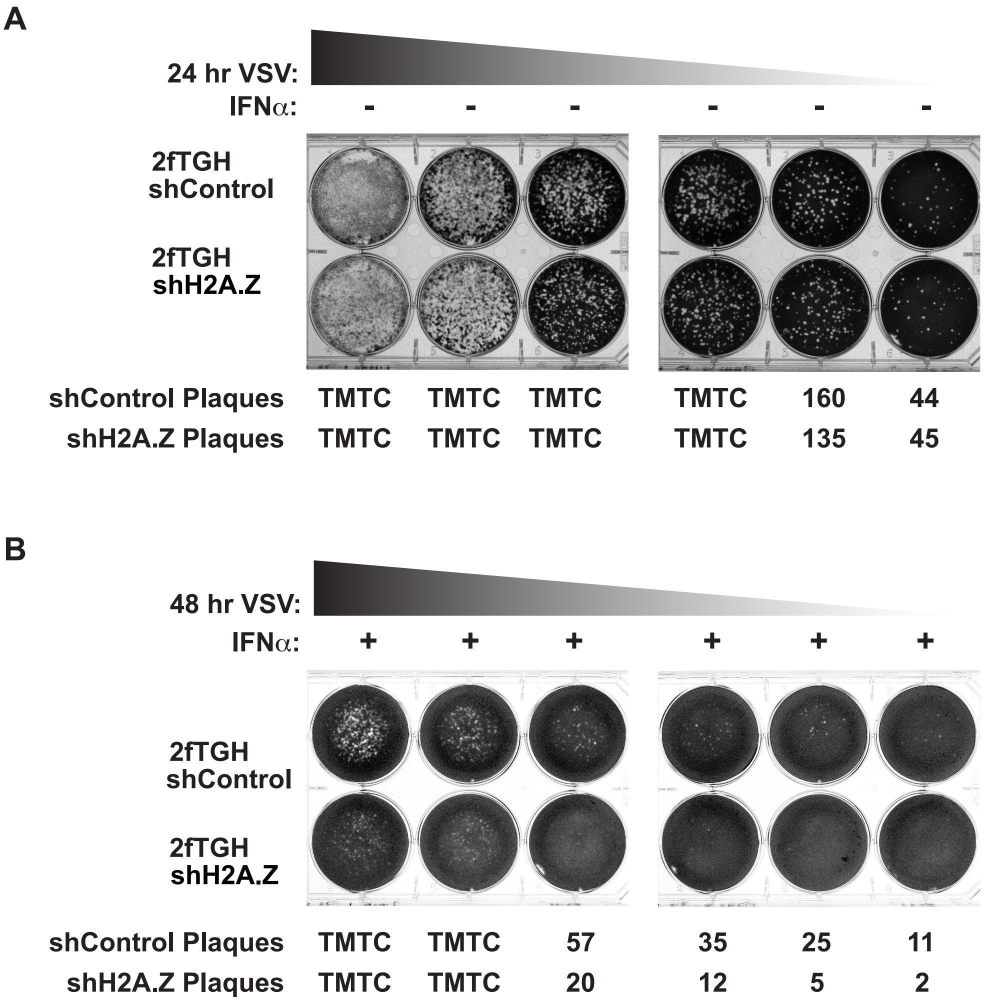

Figure S4. Loss of H2A.Z does not alter VSV replication, Related to Figure 7
(A) Plaque assay in 2fTGH cells harboring shRNA non-targeting control or H2A.Z shRNA. Cells were inoculated with a VSV titration for 1.5h as in Figure 7F, but with no IFN stimulation, and overlaid with 2% DMEM-agar at 37ºC for 24h before staining with crystal violet. (B) Plaque assay of 2fTGH cells harboring shRNA non-targeting control or H2A.Z shRNA. Cells were treated for 9h with IFNα before inoculation with VSV as in Figure 7F, and incubated for 48h before staining with crystal violet. TMTC, too many to count.

A

Gene	Known ISGF3 Targets	Cluster		Locus	Gene Size (bp)	Total Nucleosomes	Nucleosomes per bp
IFIT1 (ISG56)	✓			chr10:91,152,322-91,163,742	11,420	64	0.0056
IFIT2 (ISG54)	✓	✓		chr10:91,061,706-91,069,032	7,326	35	0.0048
IFIT3 (ISG60)	✓			chr10:91,092,239-91,100,724	8,485	47	0.0055
IFIT5				chr10:91,174,325-91,180,758	6,433	21	0.0033
ISG15	✓			chr1:948,847-949,915	1,068	6	0.0056
IFITM1 (9-27)	✓			chr11:313,991-315,271	1,280	6	0.0047
IFITM2		✓		chr11:308,107-309,409	1,302	9	0.0069
IFITM3 (1-8U)	✓			chr11:319,673-320,914	1,241	10	0.0081
IFITM5				chr11:298,203-299,526	1,323	7	0.0053
OAS1	✓			chr12:113,344,739-113,357,711	12,972	66	0.0051
OAS2	✓	✓		chr12:113,416,274-113,449,527	33,253	172	0.0052
OAS3	✓			chr12:113,376,249-113,411,052	34,803	177	0.0051
MX1	✓	✓		chr21:42,797,978-42,831,140	33,162	165	0.0050
MX2	✓			chr21:42,733,950-42,780,869	46,919	227	0.0048
STAT1				chr2:191,833,762-191,878,976	45,214	222	0.0049
STAT2				chr12:56,735,384-56,753,909	18,525	85	0.0046
IFI6 (6-16)	✓			chr1:27,992,572-27,998,724	6,152	23	0.0037
AIM2		✓		chr1:159,032,275-159,046,647	14,372	61	0.0042
IFI16				chr1:158,979,682-159,024,943	45,261	204	0.0045
IFI27	✓			chr14:94,577,079-94,583,033	5,954	30	0.0050

B

	Total reads	Unique reads	137-157 bp reads	Read coverage (fold)
Mock	104,796,614	55,463,526	6,720,135	562
2 hr IFNα	106,756,227	56,949,366	4,302,688	360
6 hr IFNα	94,274,867	51,243,148	5,050,067	422
10 hr IFNα	103,862,266	55,437,595	7,280,912	608

Table S2. Direct selection nucleosome selection criteria and sequencing analysis, Related to Figure 2

(A) Summary of the rationale in selecting 20 representative ISGs for nucleosome profiling and characterization of the ISG nucleosome composition (total nucleosomes, average nucleosome per bp). (B) Total and unique sequencing reads from each sample and the read enrichment calculated from (number of reads x 147 bp)/(1758135 bp length of BACs).

Table S3. Reagents, qPCR Primers and Plasmids, Related to Figures 1, 2, 3, 4, 5, 6, 7, S1, S2, S3

mRNA/cDNA RT-PCR Primers

	Forward Primer	Reverse Primer
H2A.Z	CTCACCGTGGGTCCGATTAG	CGCCTTTGTCTTGGCCTTTC
OAS3	TTCATCCAGGACCACCTGA	GCCAAATGAGCCCCCTTTAC
IFIT1/ISG56	CAGAACGGCTGCCTAATTT	GGCCTTTCAGGTGTTTCAC
IFIT2/ISG54	GGAAGATTTCTGAAGAGTGCAG	CTCCCTCCATCAAGTTCCAG
IFITM1/9-27	CCTTCCAAGGTCCACCGT	ACGTCGCCAACCATCTTC
LOC100419583	GCTTGCTCAGGTCTCTGTCC	CTGCCCGGTAGTTATTCAGC
ISG15	GACCTGACGGTGAAGATGCT	CGATCTTCTGGGTGATCTGC
GAPDH	ACAGTCAGCCGCATCTTCTT	ACGACCAAATCCGTTGACTC

Genomic DNA/ChIP RT-PCR Primers

	Forward Primer	Reverse Primer
OAS3 promoter	CAAGTTTGGGGAAGACAGGA	TCGGATTTCTGGTTTCGTTT
OAS3 gene body	AAAGCCAGCCAGTGAACAGT	ATCCAAGCCACTCTCCTCAA
IFIT1/ISG56 promoter	GCAGGAATTCCGCTAGCTTT	GCTAAACAGCAGCCAATGGT
IFIT1/ISG56 gene body	CCTCCTTGGGTTCGTCTACA	GGCTGATATCTGGGTGCCTA
IFITM1/9-27 promoter	CAGCAGGAAATAGAAACTTAAGAGAAA	GGGGAAGGAAGTGTTGAGTG
IFITM1 /9-27 gene body	CTGATTCTGGGCATCCTCAT	AGGCTATGGGCGGCTACTA
LOC100419583 promoter	TTGCTGATCTCATCACTGCAT	ACTTTCCCGTCCTGGTTTCT
ISG15 promoter	CGTGTGTGCCTCAGGCTTAT	ACGGCACAAGCTCCTGTACT
IFIT3/ISG60 promoter	ATTTTCCTCCTCCCAACGAT	GAGAGTAGGGCACGCATCAG
βActin promoter	CTGGGTTCTGTACGCTCCTG	GACCCACCCAGCACATTTAG

Plasmids or siRNA

	Sequence	Clone ID
shRNA: H2AFZ	CGTATTCATCGACACCTAA	V2LHS_132986
shRNA: H2AFZ	GCCGTATTCATCGACACCT	V2LHS_132984

shRNA: H2AFZ	CCGTATTCATCGACACCTA	V2LHS_132985
shRNA: Non-silencing	N/A	RHS 4346
shRNA: INO80	ATTTCTTCCAGTACAGAAG	V2LHS_238013
shRNA: RVB1	TTAGCAAGCAAGTTGGCCG	V2LHS_14740
shRNA: RVB2	TGCTGGTCGATCAATCTGG	V3LHS_641743
shRNA: CBP	TAAGTGATAATATTCATCC	V2LHS_24251
siRNA: SRCAP	Dharmacon On-TARGETplus SMARTpool	L-004830-00-0005

Bacterial artificial chromosomes (BAC)

BAC	BAC start-end	Hybridization ISG target(s)
RP11-1065J8	Chr1: 158892666- 159066838	IFI16, AIM2
RP11-1107P24	Chr10: 91017523- 91231100	IFIT1, IFIT2, IFIT3, IFIT5
RP11-932J23	Chr12: 113297537- 113465374	OAS1, OAS2, OAS3
RP11-120C17	Chr21: 42708733- 42858453	MX1, MX2
CTD-2344F1	Chr11: 212684- 355505	IFITM1, IFITM2, IFITM3, IFITM5
CTD-3113J13	Chr1: 838835- 1031922	ISG15
RP11-553K16	Chr1: 27887559-28085894	IFI6
RP11-641G12	Chr2: 191780297- 191951049	STAT1
RP11-348M3	Chr12: 56684981- 56848839	STAT2
RP11-668H1	Chr14: 94486463- 94670429	IFI27

TRANSPARENT METHODS

Cell culture and treatment with interferon or chemical inhibitor

Human cells lines, HeLa S3, 293T/17, 2fTGH, U2A, U3A and U6A cells were grown in Dulbecco's modified Eagle's medium (DMEM) containing 10% cosmic calf serum (CCS) and 1% pen-strep (PS) at 37ºC with 5% CO_2. Lentiviral-transduced HeLa or 2fTGH cells harboring shRNA were cultured in DMEM containing 10% CCS, 1% PS and 5-10 μg/ml puromycin (Sigma, St. Louis, MO) at 37ºC with 5% CO_2. Cells were mock-treated or treated with 1000 units/ml of IFNα (Hoffman-Roche) for the specified amount of time. Chemical inhibitors, PFI-3 (0.9 mM, Sigma), TSA (1.3 mM; EMD Millipore, Burlington, MA), MB-3 (0.5 mM, Sigma), JQ1 (10 μM; Apexbio, Houston, TX), and BIC1 (0.5 mM, Sigma) were added 1h pre-IFN treatment and not removed during IFN treatment. See Table S3 and Key Resources Table for reagent details.

mRNA Expression

RNA was isolated from cells with Trizol, extracted with phenol/chloroform and isopropanol-precipitated. RNA was treated with DNase I, primed with random primers and reverse transcribed to cDNA with Superscript III (Invitrogen, Carlsbad, CA). Relative mRNA abundance was determined by SYBR green qPCR (Invitrogen) using specific primers. Analysis was based on the delta Ct method using GAPDH to normalize for relative abundance. Statistical analysis was computed using the Student's t-test with $n \geq 3$. See Table S3 and Key Resources Table for reagent details.

Lentivirus-mediated RNA interference

293T/17 cells were transfected with pGIPZ lentiviral short hairpin RNA vectors, pΔ8.91 and pUC-MDG using either lipofectamine 2000 or polyethylenimine in DMEM media for 15h followed by a change to DMEM media supplemented with 10% CCS and 1% PS for 24h. Lentivirus-containing supernatant was centrifuged and filtered to remove cell debris. HeLa or 2fTGH cells were transduced 2-3 times with fresh lentivirus and polybrene for 24h each time. See Table S3 and Key Resources Table for reagent details.

siRNA-mediated RNA interference

HeLa or 2fTGH cells were transfected with 40 nM siRNA using lipofectamine 2000 for 48 hr. Cells were mock- or IFN-treated and harvested by Trizol for RNA analysis or processed for ChIP sample preparation. See Table S3 and Key Resources Table for reagent details.

Antiviral plaque assay

Lentivirus-shRNA-transduced HeLa or 2fTGH cells were mock-treated or treated with 1000 units/ml of IFNα for the specified amount of time, then infected with vesicular stomatitis virus (Indiana strain) for 24-72h at 37ºC with 5% CO_2. Cells were fixed with 3.7% formaldehyde and stained with crystal violet.

Immunoblot

Cells were lysed on ice in whole cell extract buffer (50 mM Tris pH 8.0, 280 mM NaCl, 0.5% Igepal, 0.2 mM EDTA, 2 mM EGTA, 10% glycerol) supplemented with fresh DTT, protease inhibitor and sodium vanadate for 15-30 min, then sonicated for 5 minutes (15 sec on, 45 sec off) at 4°C using a cuphorn sonicator (Misonix). The sonicated sample was centrifuged at 14,000 x g for 15 minutes to remove the cellular debris. Total protein was denatured at 100°C in SDS loading buffer, separated by SDS-PAGE, transferred to nitrocellulose membrane, blocked in milk/TBST solution, probed with specific antibody (H2A.Z ab4174 Abcam, Cambridge, MA; GAPDH sc-47724 Santa Cruz, Dallas, TX; STAT1 Santa Cruz sc-345, STAT2 sc-476) and the corresponding HRP

conjugated secondary antibody (Invitrogen). Chemiluminescent detection (PerkinElmer, Waltham, MA) was performed using Vision Works software. Relative density was quantified using ImageJ software. See Table S3 and Key Resources Table for reagent details.

ChIP sample preparation

Chromatin immunoprecipitation (ChIP) samples were prepared according to Lee et al., 2006. Adherent cells were crosslinked for 10 minutes with 11% formaldehyde solution (50 mM HEPES-KOH pH 7.5, 100 mM NaCl, 1 mM EDTA pH 8.0, 0.5 mM EGTA pH 8.0, 11% formaldehyde) and quenched for 5-10 minutes with glycine at room temperature. Crosslinked cells were lysed at 4ºC with lysis 1 buffer (50 mM HEPES-KOH pH 7.5, 140 mM NaCl, 1 mM EDTA, 10% glycerol, 0.5% NP-40, 0.25% Triton X-100, 1X protease inhibitors), pelleted to isolate the nuclei, washed with lysis 2 buffer (10 mM Tris-HCl pH 8.0, 200 mM NaCl, 1 mM EDTA, 0.5 mM EGTA, 1x protease inhibitors) and pelleted. Nuclei was resuspended in lysis buffer 3 (10 mM Tris-HCl pH 8.0, 100 mM NaCl, 1 mM EDTA, 0.5 mM EGTA, 0.1% Na-Deoxycholate, 0.5% N-lauroylsarcosine, 1X protease inhibitiors) and sonicated at 4ºC with intervals of 15 seconds on and 45 seconds off until DNA fragments were ≤1000 bp. Triton-X was added to the sonicated lysate to a final concentration of 1% and centrifuged to pellet the cell debris. The cleared lysate was removed and 1% input sample was saved. The lysate was incubated with Dynabeads bound to antibody (STAT1 Santa Cruz sc-345, STAT2 Santa Cruz sc-476, IRF9 Santa Cruz sc-496, RNA Pol II CTD Abcam ab817, H2A.Z Abcam ab4174, H2A Abcam ab18255, H2B Abcam ab1790, H3 Abcam ab1791, H4 Abcam ab7311) overnight at 4ºC followed by 5 times wash with cold RIPA buffer (50 mM HEPES pH 7.5, 500 mL LiCl, 1 mM EDTA, 1% NP-40, 0.7% Na-Deoxycholate) and once with cold Tris-EDTA (TE) pH 8.0 + NaCl. Immunoprecipitated complexes were eluted off the beads with 30 minutes of 65ºC water bath incubation and periodic vortexing. The eluate was further incubated in a 65ºC heated incubator for 12-15h. TE buffer was added to the reverse-crosslinked sample and incubated with RNase A for 2h at 37ºC, Proteinase K for 2h at 55ºC and isolated with phenol:chloroform:isoamyl. The sample was eluted with ethanol (EtOH), glycogen and NaCl at -20ºC overnight, washed with 80% EtOH and resuspended in Tris-HCl pH 8.0. ChIP DNA was used either for qPCR assays using specific primers or prepared into a sequencing library for Applied Biosystems (ABI, Foster City, CA) SOLiD 5500xl sequencing. See Key Resources Table for reagent details.

ChIP library preparation for SOLiD 5500xl sequencing

The purified ChIP DNA was prepared for sequencing following ABI SOLiD 5500xl library preparation protocol. Purified DNA was end-repaired (NEBNext; NEB, Ipswich, MA). The DNA was size-selected with Ampure beads followed by addition of a single dA-tail to the ends. ABI SOLiD 5500xl DNA adaptor barcodes were ligated onto the DNA with Quick Ligase (NEB) for 30 minutes at room temperature and size-selected with Ampure beads. Size-selected DNA was pseudo nick-translated to fill in the 5' overhang and remove the 3' end (NEBNext). Ampure beads was used to purify the final adaptor-ligated ChIP DNA. The adaptor-ligated ChIP DNA library was amplified for 11-13 cycles using the SOLiD P1 and P2 primers and DNA size was verified by Bioanalyzer. See Key Resources Table for reagent details.

ChIP assays, deep sequencing and data analysis

ChIP and input DNA abundance was analyzed using SYBR green qPCR for ChIP assays. ChIP DNA was normalized using the percent input method. Statistical analysis was computed using the Student's t-test with $n \geq 2$. For high-throughput sequencing, ChIP DNA was prepared following the SOLiD 5500xl library preparation protocol. Reads were aligned to the human hg19 build with Bioscope v1.3.1. The reference genome was converted to colorspace. MACS software was used to identify unique peaks. Genomic regions that were statistically enriched in the ChIP-Seq data (p-value $\leq 1 \times 10^{-5}$) relative to the control input DNA were identified by the MACS software (Zhang

et al., 2008), and represent regions bound by IFN-induced STAT1, STAT2 or IRF9. Additional data analysis was performed with the HOMER software.

Direct selection MNase nucleosome preparation, deep sequencing and data analysis
Mononucleosome DNA (mnDNA) from (~147-167 bp) was isolated, processed and sequenced as described in Freaney et al., 2014 and Yigit et al., 2013. Briefly, a sequencing library was prepared with isolated mononucleosome DNA (mnDNA) and ligated with SOLiD 5500xl adaptor DNA barcodes. The sequencing library was hybridized to 10 biotin-labeled bacteria artificial chromosomes (BACs; BACPAC Resources, Oakland, CA) to enrich for 20 target interferon-stimulated gene genomic loci. The target DNA was captured and eluted from streptavidin-conjugated beads. The eluted library was amplified for 13-15 cycles using SOLiD P1 and P2 primers and paired-end sequenced on the SOLiD 5500xl platform. See Table S3 and Key Resources Table for reagent details.

To obtain mnDNA, crude nuclei was isolated from HeLa cells. Approximately 5 x 10^7 adherent HeLa cells were pelleted, washed, and lysed with MC lysis buffer (10 mM Tris-HCl pH 7.5, 10 mM NaCl, 3 mM $MgCl_2$, 0.5% Igepal). The nuclear pellet was resuspended in micrococcal nuclease (MNase) reaction buffer (10 mM Tris-HCl pH 7.5, 10 mM NaCl, 3 mM $MgCl_2$, 1 mM $CaCl_2$, 4% Igepal, fresh 1 mM PMSF). MNase (800 units/5 x 10^7 cells) was added to the nuclear pellet and digested the chromatin for the appropriate amount of time to generate mononucleosome DNA at 25°C. The digestion reaction was terminated with addition of a stop reaction solution (10 mM EGTA, 1X PMSF, 1X PI, 1% SDS, 200 mM NaCl or 10 mM EDTA). The digested chromatin sample was treated with RNase A for 30 min at 37°C followed by a phenol/chloroform extraction to obtain the DNA. The DNA (~1.5 µg) was loaded onto a 3.5% NuSieve 3:1 agarose gel in 0.5% TBE solution to resolve the nucleosomal DNA bands. The mononucleosomal-sized DNA (mnDNA) band (~147 bp) was excised from the gel and isolated by the crush and soak method. Briefly, the gel was incubated with 3 times volume to gel of crush and soak buffer (300 mM NaOAc, 1 mM EDTA pH 8.0, 0.1% SDS) with gentle shaking at room temperature overnight. The crush and soak buffer containing the mnDNA was filtered (Amicon Ultrafree-Cl) and the filtrate was concentrated. The concentrated mnDNA was purified and used to prepare the sequencing library.

The purified mnDNA was prepared for sequencing following ABI SOLiD 5500xl library preparation protocol. Purified mnDNA was end-repaired. The DNA was size-selected (~147 bp) with Ampure beads followed by addition of a single dA-tail to the ends. ABI SOLiD 5500xl DNA adaptor barcodes were ligated onto the DNA with Quick Ligase for 30 minutes at room temperature and size-selected with Ampure beads. Size-selected DNA was pseudo nick translated to fill in the 5' overhang and remove the 3' end. Ampure beads was used to purify the adaptor-ligated mnDNA.

To enrich for mnDNA from 20 target ISG genomic regions, bacterial artificial chromosomes (BACs) encoding these regions were used to hybridize and enrich the target ISGs. The 10 biotin-dUTP-labeled BACs allows capture of ~1.76 Mb of the genome corresponding to 20 ISG loci and their surrounding genomic regions. The lyophilized biotin-dUTP-labeled BACs was resuspended with human *cot-1* DNA to reduce nonspecific repetitive DNA sequences with a mineral oil overlay, followed by denaturation at 95°C for 5 min, incubated at 65°C for 15 min, and then incubated with 5 µl of 2× hybridization buffer (1.5 M NaCl, 40 mM sodium phosphate buffer pH 7.2, 10 mM EDTA pH 8, 10× Denhardt's, 0.2% SDS) at 65°C for 6h. Then 2 µg of the adaptor-ligated mnDNA in 5 µl of dH_2O was denatured at 95°C for 5 min and incubated at 65°C for 15 min with a mineral oil overlay. The mnDNA was transferred to a tube containing the *cot-1* suppressed BACs and allowed to hybridize with the BACs at 65°C for 72h. The hybridization mixture of BAC and mnDNA

was added to pre-washed streptavidin magnetic beads in 150 µl Streptavidin bead binding buffer. Binding of the streptavidin magnetic beads with the biotin-dUTP labeled BAC hybridized with mnDNA was carried out on a rotator at room temperature for 30 min with periodic mixing. The magnetic beads were washed once with 1 ml 1× sodium-saline citrate (SSC) buffer with 0.1% SDS at 25°C for 15 min, and then three times with 1 ml 0.1× SSC buffer with 0.1% SDS. The hybridized BAC-mnDNA was eluted off the streptavidin beads with 100 µl of 0.1 M NaOH at 25°C for 10 min. The BAC-mnDNA eluate was neutralized by addition of 100 µl 1M Tris-HCl pH 7.5 and desalted through a Sephadex G-50 column. The eluted mnDNA was PCR-amplified for 13-15 cycles using the SOLiD P1 and P2 primers.

Sequencing reads were aligned with the Bowtie software v0.12.7 using the human reference genome build hg19/GR37. Aligned reads corresponding to the BAC-selected genomic coordinates were used to generate nucleosome occupancy maps using a center-weighted algorithm and selecting reads that were 137-157 bp, representing single nucleosome-protected regions (Freaney et al., 2014; Yigit et al., 2013). The occupancy scores from the center-weighted algorithm was normalized to 10 million reads to generate nucleosome occupancy maps. To identify nucleosome occupancy changes between the steady state and different time points of IFN-induced states, additional analysis was performed using the DANPOS software (Chen et al., 2013).

KEY RESOURCE TABLE

REAGENT or RESOURCE	SOURCE	IDENTIFIER
**Antibodies**		
STAT1	Santa Cruz	sc-345
STAT2	Santa Cruz	sc-476
IRF9	Santa Cruz	sc-496
H2A.Z	Abcam	ab4174
RNA Pol II CTD	Abcam	ab817
H2A	Abcam	ab18255
H2B	Abcam	ab1790
H3	Abcam	ab1791
H4	Abcam	ab7311
Phospho-STAT1 (Tyr 701)	Cell Signaling Technology	7649
Phospho-STAT2 (Tyr 689)	EMD Millipore	07-224
H2A.Z Acetyl K4, K7, K11	Abcam	Ab18262
GAPDH	Santa Cruz	sc-47724
**Bacterial and Virus Strains**		
Vesicular stomatitis virus	N/A	Indiana strain
Lentivirus (pUC-MDG, pΔ8.91, harbors pGIPz shRNA)	This paper	See Table S3 - Plasmids
**Chemicals, Peptides, and Recombinant Proteins**		
Dynabeads Protein G	Invitrogen	10004D
IFNα	Hoffmann-La Roche Inc	RO 22-8181/001
PFI-3	Sigma Aldrich	SML0939
MB-3	Sigma Aldrich	M2449
JQ1	Fisher Scientific/Apexbio	50-101-4886

BIC1	Sigma Aldrich	203830
TSA	EMD Millipore	647925
iProof HF DNA Polymerase	Biorad	172-5302
**Critical Commercial Assays**		
SOLiD library preparation: Fragment Library Core Kit	Applied Biosystems	4464412
SOLiD library preparation: Enzyme Module	Applied Biosystems	4464413
SOLiD library preparation: Barcode adapters	Applied Biosystems	4464406
SOLiD library preparation: Standard adapter kit	Applied Biosystems	4464411
NEBNext: END Repair Module	NEB	E6050L
NEBNext: dA-Tailing Module	NEB	E6053L
**Deposited Data**		
STAT1, STAT2, IRF9 ChIP-Seq data (HeLa cells)	This paper	GEO: GSE110067
H2A.Z Encode data (HeLa cells)	(ENCODE Project Consortium, 2012)	UCSC: wgEncodeEH002395; GEO: GSM1003483 https://genome.ucsc.edu/encode/
Human reference genome NCBI build 37, GRCh37	Genome Reference Consortium	http://www.ncbi.nlm.nih.gov/projects/genome/assembly/grc/human/
**Experimental Models: Cell Lines**		
Human: HeLa	N/A	N/A
Human: 2fTGH	George Stark lab, Cleveland Clinic	N/A
Human 2fTGH-derived (-/- IRF9): U2A	George Stark lab, Cleveland Clinic	U2A
Human 2fTGH-derived (-/- STAT1): U3A	George Stark lab, Cleveland Clinic	U3A
Human 2fTGH-derived (-/-STAT2): U6A	George Stark lab, Cleveland Clinic	U6A
Human: 293T	ATCC	CRL-11268
**Oligonucleotides**		
Primers for RT and ChIP qPCR	Eurofins, Invitrogen	See Table S3
**Recombinant DNA**		
pUC-MDG (VSV-G pseudotyped viral envelope)	Xiaomin Bao lab, Northwestern University	N/A
pΔ8.91 (Lentiviral Gag, Pol)	Xiaomin Bao lab, Northwestern University	N/A
pGIPZ shRNA	GE Dharmacon	See Table S3 - Plasmids
**Software and Algorithms**		

Bowtie	(Langmead et al., 2009)	https://sourceforge.net/projects/bowtie-bio/files/bowtie/0.12.7/
MACS	(Zhang et al., 2008)	http://liulab.dfci.harvard.edu/MACS/
DANPOS	(Chen et al., 2013)	https://sites.google.com/site/danposdoc/
HOMER	(Heinz et al., 2010)	http://homer.ucsd.edu/homer/
R	R Development Core Team	https://www.r-project.org/
**Other**		
Galaxy	(Afgan et al., 2016)	Usegalaxy.org

DATA AND SOFTWARE AVAILABILITY

All deep sequencing data have been deposited in GEO under accession number GSE110067.

SUPPLEMENTAL REFERENCES

Afgan, E., Baker, D., van den Beek, M., Blankenberg, D., Bouvier, D., Cech, M., Chilton, J., Clements, D., Coraor, N., Eberhard, C., *et al.* (2016). The Galaxy platform for accessible, reproducible and collaborative biomedical analyses: 2016 update. Nucleic Acids Res *44*, W3-W10.

Chen, K., Xi, Y., Pan, X., Li, Z., Kaestner, K., Tyler, J., Dent, S., He, X., and Li, W. (2013). DANPOS: dynamic analysis of nucleosome position and occupancy by sequencing. Genome Res *23*, 341-351.

ENCODE Project Consortium (2012). An integrated encyclopedia of DNA elements in the human genome. Nature *489*, 57-74.

Freaney, J.E., Zhang, Q., Yigit, E., Kim, R., Widom, J., Wang, J.P., and Horvath, C.M. (2014). High-density nucleosome occupancy map of human chromosome 9p21-22 reveals chromatin organization of the type I interferon gene cluster. J Interferon Cytokine Res *34*, 676-685.

Heinz, S., Benner, C., Spann, N., Bertolino, E., Lin, Y.C., Laslo, P., Cheng, J.X., Murre, C., Singh, H., and Glass, C.K. (2010). Simple combinations of lineage-determining transcription factors prime cis-regulatory elements required for macrophage and B cell identities. Mol Cell *38*, 576-589.

Langmead, B., Trapnell, C., Pop, M., and Salzberg, S.L. (2009). Ultrafast and memory-efficient alignment of short DNA sequences to the human genome. Genome Biol *10*, R25.

Yigit, E., Zhang, Q., Xi, L., Grilley, D., Widom, J., Wang, J.P., Rao, A., and Pipkin, M.E. (2013). High-resolution nucleosome mapping of targeted regions using BAC-based enrichment. Nucleic Acids Res *41*, e87.

Zhang, Y., Liu, T., Meyer, C.A., Eeckhoute, J., Johnson, D.S., Bernstein, B.E., Nusbaum, C., Myers, R.M., Brown, M., Li, W., *et al.* (2008). Model-based analysis of ChIP-Seq (MACS). Genome Biol *9*, R137.
